# Abnormal ER quality control of neural GPI-anchored proteins via dysfunction in ER export processing in the frontal cortex of elderly subjects with schizophrenia

**DOI:** 10.1038/s41398-018-0359-4

**Published:** 2019-01-16

**Authors:** Pitna Kim, Madeline R. Scott, James H. Meador-Woodruff

**Affiliations:** Department of Psychiatry and Behavioral Neurobiology, University of Alabama at Birmingham, Birmingham, AL 35294 USA

## Abstract

Abnormalities of posttranslational protein modifications (PTMs) have recently been implicated in the pathophysiology of schizophrenia. Glycosylphosphatidylinositols (GPIs) are a class of complex glycolipids, which anchor surface proteins and glycoproteins to the cell membrane. GPI attachment to proteins represents one of the most common PTMs and GPI-associated proteins (GPI-APs) facilitate many cell surface processes, including synapse development and maintenance. Mutations in the GPI processing pathway are associated with intellectual disability, emphasizing the potential role of GPI-APs in cognition and schizophrenia-associated cognitive dysfunction. As initial endoplasmic reticulum (ER)-associated protein processing is essential for GPI-AP function, we measured protein expression of molecules involved in attachment (GPAA1), modification (PGAP1), and ER export (Tmp21) of GPI-APs, in homogenates and in an ER enriched fraction derived from dorsolateral prefrontal cortex (DLPFC) of 15 matched pairs of schizophrenia and comparison subjects. In total homogenate we found a significant decrease in transmembrane protein 21 (Tmp21) and in the ER-enriched fraction we found reduced expression of post-GPI attachment protein (PGAP1). PGAP1 modifies GPI-anchors through inositol deacylation, allowing it to be recognized by Tmp21. Tmp21 is a component of the p24 complex that recognizes GPI-anchored proteins, senses the status of the GPI-anchor, and regulates incorporation into COPII vesicles for export to the Golgi apparatus. Together, these proteins are the molecular mechanisms underlying GPI-AP quality control and ER export. To investigate the potential consequences of a deficit in export and/or quality control, we measured cell membrane-associated expression of known GPI-APs that have been previously implicated in schizophrenia, including GPC1, NCAM, MDGA2, and EPHA1, using Triton X-114 phase separation. Additionally, we tested the sensitivity of those candidate proteins to phosphatidylinositol-specific phospholipase C (PI-PLC), an enzyme that cleaves GPI from GPI-APs. While we did not observe a difference in the amount of these GPI-APs in Triton X-114 phase separated membrane fractions, we found decreased NCAM and GPC1 within the PI-PLC sensitive fraction. These findings suggest dysregulation of ER-associated GPI-AP protein processing, with impacts on post-translational modifications of proteins previously implicated in schizophrenia such as NCAM and GPC1. These findings provide evidence for a deficit in ER protein processing pathways in this illness.

## Introduction

Multiple hypotheses of the pathophysiology of schizophrenia have focused on abnormalities of neurotransmission, including deficits in transmitter-specific signaling as well as in the development and maintenance of dendritic spines and synapses^1–6^. The molecular mechanisms underlying these deficits are still being defined. Recent evidence has demonstrated deficits in subcellular localization and posttranslational modifications that regulate trafficking of neurotransmitter receptors and transporters^1,7–11^. Additionally, we have previously demonstrated abnormalities in endoplasmic reticulum (ER) retention signals and proteins regulating ER quality control and degradation pathways^12,13^, implicating a deficit in ER-associated protein processing in schizophrenia. As such, we propose that ER-protein processing pathways that impact synapse development and maintenance are previously substrates underlying the cellular pathophysiology of schizophrenia.

Anchoring of proteins to the cell membrane through the addition of a glycosylphosphatidylinositol (GPI)-anchor is responsible for the activity and localization of a specialized class of lipid-associated neuronal membrane proteins that have roles in synaptic formation, neurotransmission, and synaptic plasticity^14^. GPI-anchored proteins (GPI-APs) are a particular category of luminal secretory proteins attached to the membrane by a glycolipid anchor that is post-translationally added in the ER. The GPI anchor consists of a lipid moiety with a glycan backbone and is generated by a series of sequential reactions at the ER membrane. Once synthesized, the anchor is attached to protein substrates by the GPI transamidase complex, after which it undergoes further modification within the ER. A primary modification is inositol deacylation by post-GPI attachment protein 1 (PGAP1)^15^, which is required for efficient ER export. As a quality control mechanism, the p24 complex binds to GPI-APs, senses the status of GPI-anchor modification, and regulates whether these substrates are exported to the Golgi or retained for further processing. After exiting the ER, GPI-APs are transported along the secretory pathway, through the Golgi complex, to their final destination at the plasma membrane. GPI-APs are involved in cell recognition and interaction, signal transduction, cell activation, and cell surface enzymatic reactions^16^. Recent studies have reported that a subset of GPI-APs act as modulators of synapse development in cooperation with specific synaptic adhesion pathways via direct interactions with key synapse-organizing proteins^14,17–20^.

Dysfunction in the synthesis, intracellular trafficking, and plasma membrane organization of GPI-APs have been associated with brain disorders^21^. Notably, mutations in genes that synthesize GPI anchors have been linked to several forms of intellectual disability^22–27^. This suggests a fundamental role for GPI-anchors in cognition, leading us to hypothesize that this system may be dysregulated in schizophrenia. To test this, we measured protein expression of GPI anchor attachment 1 (GPAA1), a component of the GPI transamidase complex that attaches the GPI anchor to protein substrates, post-GPI attachment protein 1 (PGAP1), which deacylates the GPI anchor, and transmembrane protein 21 (Tmp21), a subunit of the p24 complex that regulates ER export of GPI-APs, in postmortem samples of dorsolateral prefrontal cortex (DLPFC) from subjects with schizophrenia and matched comparison subjects. Abnormal expression of key regulatory protein in this system suggests dysregulation of GPI-AP proteins themselves. To test whether there are abnormalities in GPI-dependent membrane association, we identified several brain-expressed GPI-APs previously associated with schizophrenia, glypican 1 (GPC1)^28,29^, neural cell adhesion molecule 1 (NCAM)^30,31^, MAM domain-containing GPI protein 2 (MDGA2)^32^, and ephrin type A receptor (EPHA1)^33^. For these target proteins, we compared the proportion of each associated with the cell membrane and sensitive to treatment with phosphatidylinositol-specific protein lipase C (PI-PLC), an enzyme that specifically cleaves the GPI-anchor, in schizophrenia and comparison subjects. These studies characterize abnormalities of GPI-AP quality control proteins in the DLPFC in schizophrenia, and begin determine to the impact of their dysregulation on post-translational modification of proteins previously associated with this illness.

## Materials and methods

### Human subjects

Samples of dorsolateral prefrontal cortex (Brodmann Areas 9/46) were obtained from the Mount Sinai/Bronx Veterans Administration Medical Center Department of Psychiatry Brain Collection. Assessment, consent and postmortem procedures were conducted as required by the Institutional Review Boards of Pilgrim Psychiatric Center, Mount Sinai School of Medicine and the Bronx Veterans Administration Medical Center^34,35^. Subjects diagnosed with schizophrenia based on DSM-III-R criteria were pairwise matched with comparison subjects by sex, age, tissue pH, and postmortem interval (PMI) (Table 1 and Supplementary Table S1). The majority of subjects are the same between all experiments, with a few subjects re-paired due to tissue constraints (Supplementary Table S1). Brains from subjects were removed at autopsy and sliced into 0.8–1 cm slabs in the coronal plane. Full thickness of gray matter from the cortex was blocked into 1 cm^3^ cubes and stored at −80 °C until use^34,35^.

**Table 1:** Summary of subject demographics

	Comparison	Schizophrenia
*n*	15	15
Age	77.07 ± 10.02	77.80 ± 10.34
Sex	8 M/7 F	8 M/7 F
PMI (h)	8.01 ± 6.37	15.36 ± 6.02
Tissue pH	6.55 ± 0.32	6.51 ± 0.21
On/Off Rx	0/15	3/12

*PMI* postmortem interval, Rx: *On* treated with antipsychotic medications at time of death, *Off* off antipsychotic medication for ≥6 weeks prior to death

### Antipsychotic-treated tats

Male Sprague-Dawley rats were housed in pairs and treated with chronic administration of either 28.5 mg/kg haloperidol decanoate (HAL; *N* = 10) or vehicle (VEH; *N* = 10) delivered once every 3 weeks for 9 months via intramuscular injection, for a total of 12 injections. This method of drug delivery, length of treatment, and dose has been previously described^36–38^. Animals were euthanized by rapid decapitation following CO_2_ administration, frontal cortexes were dissected on ice, then snap frozen and stored at −80 °C until use. The Institutional Animal Care and Use Committee of the University of Alabama at Birmingham approved all procedures using these animals.

### Effect of postmortem interval in mice

To determine the effects of PMI on protein expression in mouse brain, we modeled PMI conditions typically seen at the time of death in humans. Fifteen female C57BL/6 mice were used for this study. Brains were removed and kept at 4 °C. Following decapitation, Hour 0 samples (*N* = 3) were immediately frozen in dry ice, and then stored at −80 °C. Remaining brains were held at 4 °C for progressively longer times (1 h, 4 h, 10 h, *N* = 4 at each time point), and then transferred to −80 °C for storage. The frontal cortex was dissected from each brain for western blot analyses. This study was approved by the Institutional Animal Care and Use Committee of the University of Alabama at Birmingham.

### Tissue preparation

Tissue samples from human subjects (50 mg wet weight) were pulverized using a mortar and pestle with the addition of small amounts of liquid nitrogen. Isotonic extraction buffer (10 mM HEPES, pH 7.8; 250 mM sucrose; 25 mM potassium chloride; 1 mM EGTA) was added to each sample and tissues homogenized with 10–12 strokes in a glass-teflon homogenizer before being transferred to a nitrogen cavitation chamber (Parr Instrument Company, Moline, IL, USA). Homogenates were pressurized to 450 psi for 8 min and collected through the outlet port of the chamber by nitrogen decompression.

DLPFC samples for phosphoinositide-specific phospholipase C (PI-PLC) enzyme treatment and Triton X-114 phase partitioning, as well as samples obtained from antipsychotic treated rats, were suspended in sucrose homogenization buffer (320 mM sucrose in 5 mM Tris-HCL, pH 7.5) containing with protease and phosphatase inhibitor tablets (Complete Mini, EDTA-free and PhosSTOP, Roche Diagnostics, Indianapolis, IN) and homogenized with 10–12 strokes in a glass-teflon homogenizer. Protein concentrations of all samples were assessed by BCA assays (Thermo Fisher Scientific, Rockford, IL, USA) and samples were stored at −80 °C until use.

### Subcellular fractionation

Subcellular fractionation was performed in parallel for each pair of subjects as previously described^39,40^. 60 µL aliquots of nitrogen cavitated tissue were reserved as the total homogenate sample for western blot analyses. The remainder of each sample was then centrifuged at 700×*g* for 10 min at 4 °C, followed by a 15,000 × *g* centrifugation of the resulting supernatant. The supernatant from the second centrifugation step was then layered onto a sucrose gradient consisting of 1 mL of each 2.0 M, 1.5 M, and 1.3 M sucrose in a 14 × 89 mm Beckman polyallomer ultracentrifuge tube. Sucrose gradients were ultracentrifuged at 126,000 × *g* (35,000 rpm in a SW60 Ti rotor) at 4 °C for 70 min. A dense white band at the interface of the 1.3–1.5 M sucrose layers (~100–300 μL) was extracted and combined with cold 1× MTE buffer (270 mM D-mannitol; 10 mM Tris-base; and 0.1 mM EDTA, pH 7.4) and phenylmethylsulfonyl fluoride (PMSF, 1 mM), and ultracentrifuged at 126,000 × *g* for 45 min at 4 °C to obtain a pellet enriched for ER membranes. This pellet was resuspended in 50 µL of 1× phosphate buffered saline (PBS), pH 7.4, and protein concentration was determined by BCA protein assay (Thermo Fischer Scientific).

### Western blot for protein expression

Samples for western blot analyses were prepared with Milli-Q water and sample buffer (6× solution: 4.5% sodium dodecyl sulfate (SDS), 15% β-mercaptoethanol, 0.018% bromophenol blue, and 36% glycerol in 170 mM Tris-HCl, pH 6.8) and heated at 70 °C for 10 min. Samples were loaded on 4–12% Bis-Tris gels (Invitrogen) then subjected to SDS-polyacrylamide gel electrophoresis (SDS-PAGE) and transferred to nitrocellulose membranes using standard semi-dry transfer methods. To reduce non-specific binding, blots were incubated in either 50% Odyssey Blocking buffer (Li-Cor) in Tris-buffered saline (TBS) or 5% bovine serum albumin in TBS for 1 h at room temperature (RT) prior to primary antibody incubation. Conditions for probing were optimized to be within the linear range of detection for each primary antibody (Supplementary Table S2). Tris-buffered saline (TBS) + 0.1% Tween-20 was used for wash steps before and after a 1 h incubation at RT with the appropriate secondary antibody, and membranes rinsed with Milli-Q water prior to scanning. Membranes were scanned using a LiCor Odyssey scanner at a resolution of 169 µm and intensity level of 5.

### PI-PLC enzyme treatment and phase partitioning with Triton X-114

PI-PLC specifically cleaves the phosphatidylinositol component of GPI anchors, effectively separating the substrate protein from its membrane-integrated lipid tether. To disrupt the interaction between GPI-APs and cell membranes, samples were treated with PI-PLC and then Triton X-114 phase partitioned. Triton X-114 partitioning produces two distinct phases: an aqueous phase (AQ), which contains cytosolic and extracellular components that are not bound to or positioned within lipid membranes, and a detergent phase (DT), which contains lipid-rich membranes, transmembrane proteins, and other proteins that are bound to membranes or integrated into membrane-bound complexes. In their normal physiological state, GPI-APs primarily partition to the detergent phase. After treatment with PI-PLC, however, GPI-APs are released from the membrane and partition into the aqueous phase.

For PI-PLC treatment, 5 μL of PI-PLC (Millipore Sigma, St. Louis, MO, USA) was added to 500 μL of tissue homogenate (0.2 μg/μL) and incubated for 1 h at 37 °C. Non-enzyme-treated control samples were processed in parallel with Milli-Q water substituted for the enzyme. For each PI-PLC-treated and control sample, 100 μl was reserved to load as “input” on western blots. For phase partitioning, 100 μL of Triton X-114 was added to each sample, briefly vortexed, then incubated at 37 °C for 1 h until a clear interface was visible. The top aqueous layer was transferred to a new tube and ice-cold PBS was added to each phase to bring the sample volume to 800 μL. Next, 700 μL of acetone (−20 °C) was added to each sample and stored overnight at −20 °C. Samples were then centrifuged at 15,000 × *g* at 4 °C for 30 min. Supernatant were discarded and pellets resuspended with 50 μL of PBS containing protease inhibitor tablets (Roche). Protein concentrations were determined by BCA assays (Thermo Fischer). Samples were stored at −20 °C until processed for western blot analyses.

### Data and statistical analysis

Odyssey 3.0 analytical software (LiCor, Lincoln, NE, USA) was used to determine integrated intensity values from western blots. For whole cortical homogenates, integrated intensity values were normalized to intralane values of Valosin Containing Protein (VCP), which has been reported to not be differentially expressed in schizophrenia brain^41^. In ER-enriched fractions, integrated intensity values were normalized to intralane JM4, an ER resident protein that has uniform expression in this ER-enriched fraction^8,42^. Before target protein normalization, we verified that VCP and JM4 were not differently expressed between diagnostic groups in the total homogenate or subcellular fractions, respectively. All samples were run in duplicate and after intra-lane normalization the average for each subject was determined. To calculate enzymatic action of PI-PLC treatment on GPI-APs, we used the formula: [(average protein of interest (POI) expression before PI-PLC treatment) − (average POI expression after PI-PLC treatment)]/total POI expression in DT phase]. Total POI expression was calculated as the sum of the averaged POI expression in DT and AQ phases. To determine if data were normally distributed, D’Agostino & Pearson omnibus normality tests were performed. Normally distributed data were analyzed using paired Student’s *t*-tests, and non-normally distributed data were analyzed using Wilcoxon matched pairs signed rank tests. To reduce the risk of type I statistical errors associated with multiple comparisons, we used the Benjamini–Hochberg method^43^. Briefly, *p*-values are ranked and used to calculate the Benjamini-Hochberg critical value *q* = ([(individual *p*-value rank)/(total number of tests)]/false discovery rate (FDR)). In this study, we used an FDR of 0.2 which was calculated by [(cutoff *p*-value) × (number of probe sets tested)/(total number of positives)]^44,45^. Tests where the original *p*-value was less than associated *q*-values were considered significant. Correlations between protein expression and subject age, tissue pH, and PMI were performed for all dependent measures using Pearson correlation coefficients (*r*) calculated for normally distributed data and Spearman rank correlation coefficients (*ρ*) calculated for data not normally distributed. If any correlations were found, an ANCOVA was then used to determine the effect of diagnosis when accounting for the relevant covariates. The effect of PMI on protein expression in mice was assessed using a one-way ANOVA. Statistica (StatSoft, Tulsa, Oklahoma) and Prism 7.0 (GraphPad Software, La Jolla, CA) were used for all statistical analyses, with *α* = 0.05.

## Results

### Reduced Tmp21 protein expression in homogenate and PGAP1 protein expression in an ER-enriched fraction in schizophrenia

**Fig. 1:**
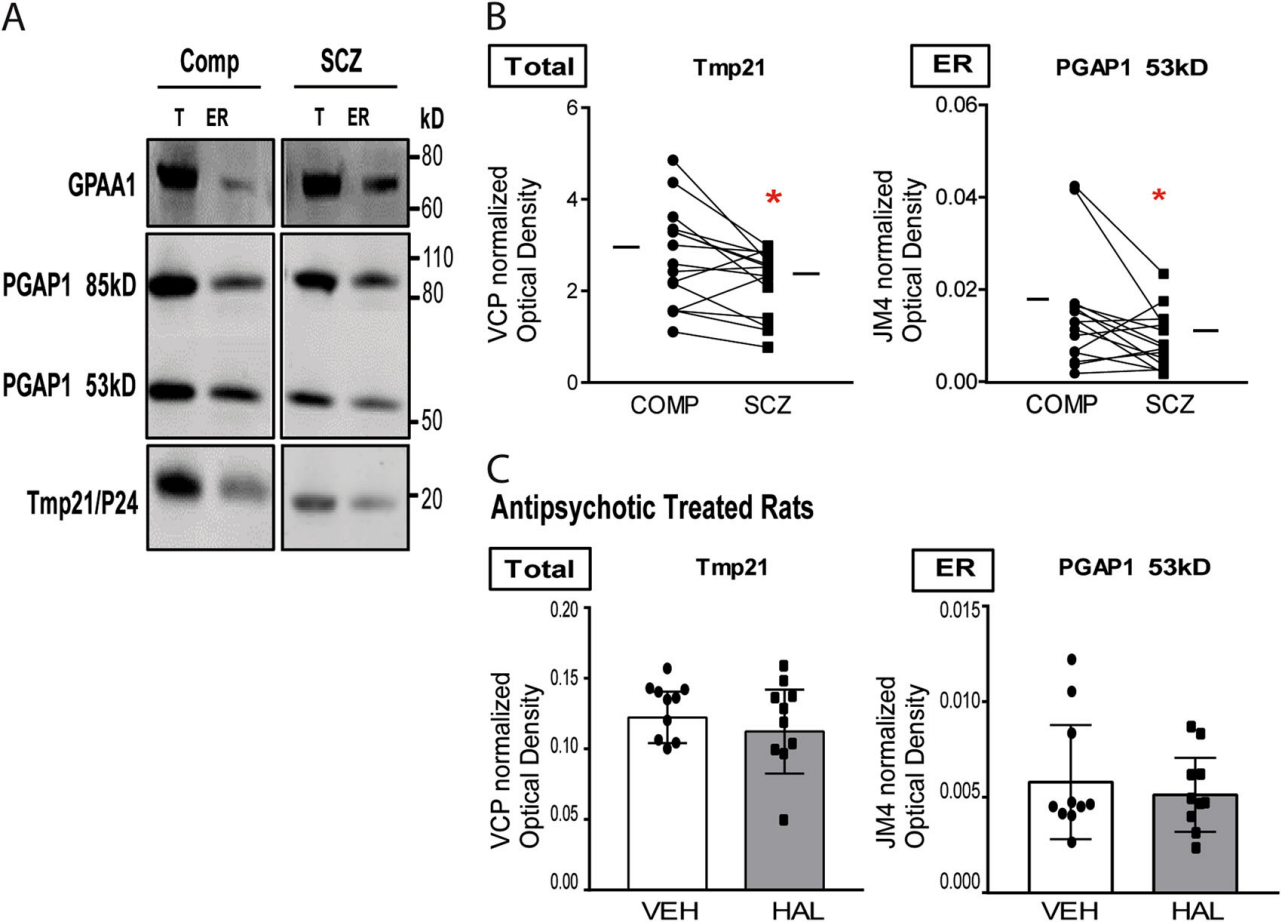
Abnormal protein expression of ER exit pathway components in schizophrenia

We examined protein expression in DLPFC homogenate of GPAA1, PGAP1, and Tmp21. We found decreased protein expression in schizophrenia of Tmp21 [*t*(14) = 2.56, *p* = 0.023], but no difference in GPAA1 or PGAP1 expression (Fig. 1a, b, Table 2). Additionally, we measured these same proteins in an ER-enriched fraction derived from DLPFC. PGAP1 has been reported as having 4 isoforms ranging from 49 to 105 kD due to alternative splicing. In DLPFC, we found that PGAP1 appears primarily as 2 bands on western blot analysis, with molecular masses of 53 and 85 kD. In an ER enriched fraction, we observed decreased expression of PGAP1 (53 kD) [t (14) = 2.17, p = 0.047] in schizophrenia (Fig. 1a, b). Neither homogenate, nor ER-enriched fraction associated expression of these proteins was significantly correlated with age, pH, or PMI, nor was there a sex effect.

**Table 2:** Expression of ER exit pathway proteins in schizophrenia and matched comparison subjects

Total					
Target protein	Comparison	Schizophrenia	Statistic	*p*	*q*
GPAA1	1.2 ± 0.69	1.0 ± 0.49	*t*(14) = 0.69		
Tmp21	3.0 ± 1.2	2.4 ± 0.80	*t*(14) = 2.6	0.023	0.025
PGAP1 85kD	0.0067 ± 0.0041	0.0092 ± 0.014	*t*(14) = 0.80		
PGAP1 53kD	0.030 ± 0.019	0.041 ± 0.018	*W* = −40		
ER					
Target protein	Comparison	Schizophrenia	Statistic	*p*	*q*
GPAA1	0.16 ± 0.18	0.24 ± 0.27	*W* = 44		
Tmp21	27 ± 15	22 ± 15	*t*(14) = 0.70		
PGAP1 85kD	0.019 ± 0.027	0.0056 ± 0.0047	*W* = −58		
PGAP1 53kD	0.017 ± 0.015	0.0099 ± 0.0077	*t*(14) = 2.2	0.047	0.050

Values are reported as means ± SEM. For normally distributed dependent measures, data were analyzed using paired *t*-test (*t*); for dependent measures that were not normally distributed, data were analyzed using Wilcoxon matched pairs signed rank test (*W*). The Benjamini–Hochberg method was used to adjust for multiple testing, *q*-values > original *p*-values were considered significant

### PI-PLC sensitivity of schizophrenia-associated GPI-APs in the DLPFC

**Fig. 2:**
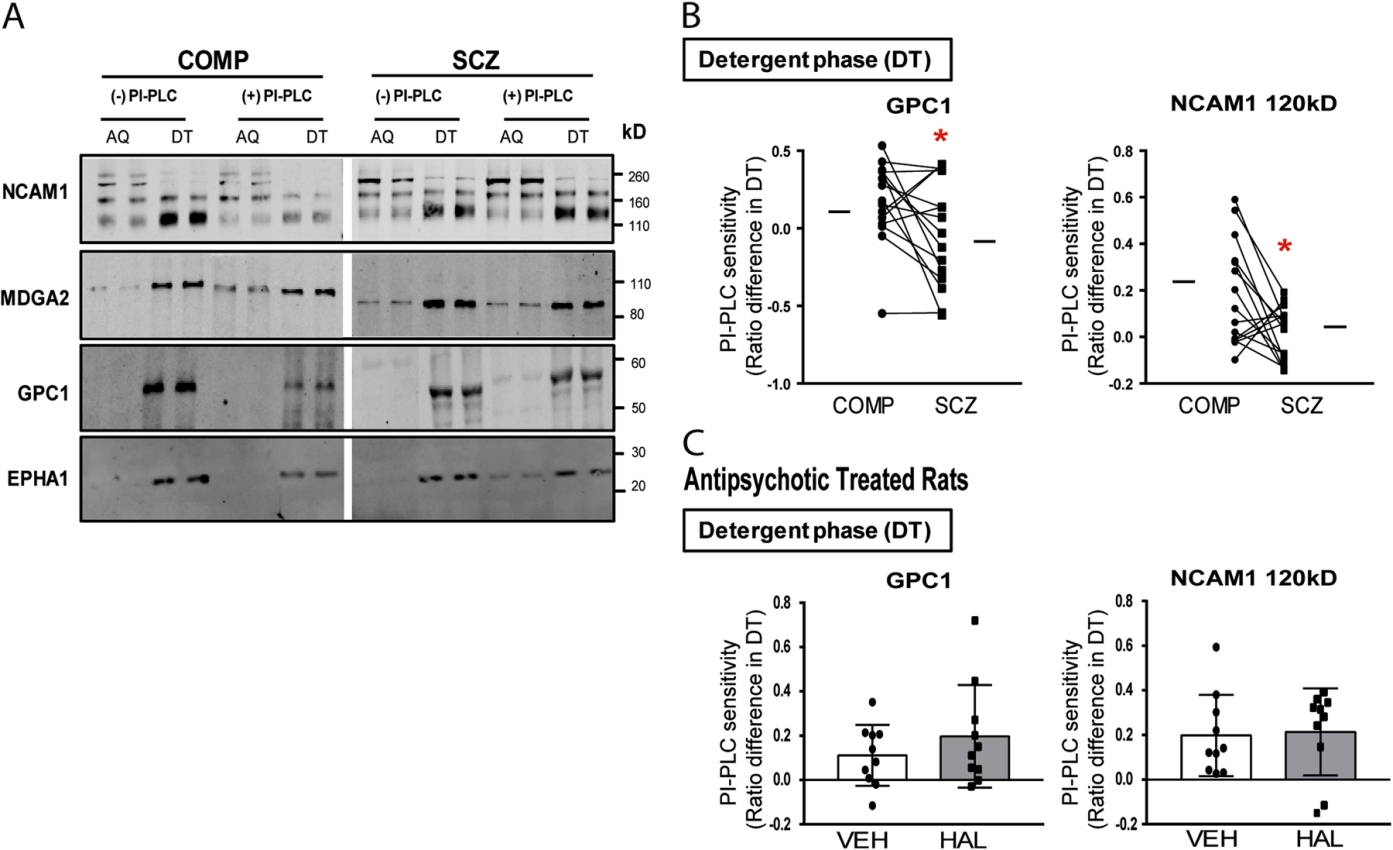
PI-PLC sensitivity of membrane-associated GPI-APs in schizophrenia

To determine potential downstream effects of abnormal Tmp21 and PGAP1 expression on GPI-APs, we investigated expression of membrane-bound GPI-APs and the sensitivity of these proteins to PI-PLC treatment. Using Triton X-114 phase separation, we isolated membrane-bound proteins in the detergent phase, while soluble forms were found in the aqueous phase. We then measured expression of four known GPI-APs (GPC1, NCAM, MDGA2, and EPHA1) in both the DT and AQ phases. DT phase expression of these GPI-APs revealed no significant difference in expression in schizophrenia (Table 3). PI-PLC-sensitivity of GPI-APs between schizophrenia and comparison subjects was also determined, with GPC1 [*t* (14) = 2.19, *p* = 0.046] and NCAM [*t* (14) = 2.57, *p* = 0.022] having decreased sensitivity to PI-PLC treatment in schizophrenia (Fig. 2a, b, Table 3).

**Table 3:** GPI-APs expression following PI-PLC treatment of Triton x-114 phase separation

Target protein	Detergent phase (DT)							
	Protein expression			PI-PLC sensitivity (ratio difference)				
	Comparison	Schizophrenia	Statistic	Comparison	Schizophrenia	Statistic	*p*	*q*
NCAM1 120 (GPI-linked form)	0.84 ± 0.17	0.85 ± 0.12	*W* = −20	0.24 ± 0.16	0.044 ± 0.13	2.6	0.022	0.034
NCAM1 140	0.70 ± 0.24	0.70 ± 0.16	0.006	0.047 ± 0.21	0.019 ± 0.17	0.38		
NCAM1 160	0.43 ± 0.24	0.38 ± 0.22	0.33	0.049 ± 0.26	0.034 ± 0.25	0.82		
MDGA2	0.43 ± 0.31	0.33 ± 0.31	0.74	0.17 ± 0.46	0.082 ± 0.37	1.6		
GPC1	0.48 ± 0.21	0.62 ± 0.21	2.1	0.11 ± 0.24	0.084 ± 0.31	2.2	0.046	0.067
EPHA1	0.75 ± 0.17	0.75 ± 0.24	0.1	0.11 ± 0.19	0.023 ± 0.31	0.99		

Data are reported as means ± SEM. For normally distributed dependent measures, data were analyzed using paired *t*-test (*t*) Student’s *t*-tests; for dependent measures that were not normally distributed, data were analyzed using the Wilcoxon matched pairs signed rank test (*W*). The Benjamini–Hochberg method was used to adjust for multiple testing, *q*-values > original *p*-values were considered significant

### Rodent models of potential confounding variables

Chronic haloperidol treatment was used to determine if antipsychotic exposure in rats was sufficient to induce the abnormalities we observed in schizophrenia. Additionally, to address possibility that protein reduction in subjects with schizophrenia may be caused by increased PMI, we modelled delays from time of death to freezer storage (0, 1, 4, and 10 h) in mice. Neither haloperidol treatment nor PMI in these rodent experiments affected protein levels of Tmp21 or PGAP1 (53 kD), suggesting that the decreased expression of Tmp21 and PGAP1 (53 kD) observed in schizophrenia is not due to these factors (Fig. 1c). Additionally, chronic antipsychotic treatment did not affect PI-PLC sensitivity of GPC1 or NCAM1 (Fig. 2c).

## Discussion

Many cell surface proteins are post-translationally modified in the ER with glycosylphosphatidylinositol (GPI). The GPI anchor is a commonly expressed protein modification that mediates localization of enzymes, receptors, differentiation antigens, and other biologically active proteins bound to the plasma membrane^46,47^. In this study, we identified abnormalities of proteins associated with the ER exit mechanism of GPI-APs in schizophrenia. In total cortical homogenates, we observed decreased Tmp21 expression in schizophrenia, while in an ER-enriched fraction we observed decreased expression of the 53 kD isoform of PGAP1. These findings suggested decreased ER export of GPI-APs, therefore we measured expression of known GPI-APs, including GPC1, NCAM, MDGA2 and EPHA 1, with and without the GPI-anchor post translational modification using Triton X-114 phase partitioning combined with PI-PLC treatment, and found that the anchored forms of GPC1 and NCAM had reduced sensitivity to PI-PLC treatment in schizophrenia. These results suggest altered sensitivities of some schizophrenia-associated GPI-APs to PI-PLC.

There are multiple steps in GPI-AP processing within the ER, including attachment of the GPI anchor to proteins, modifying the GPI anchor, and export of GPI-APs from the ER. We measured critical proteins at each of these stages to assess this system in schizophrenia. GPAA1, a component of the complex that attaches GPI anchors to proteins, was normally expressed in both total homogenate and an ER-enriched fraction, suggesting that GPI attachment is intact in schizophrenia. However, both PGAP1 and TMP21 are abnormally expressed in schizophrenia, consistent with abnormal GPI-AP processing and export.

PGAP1 is a GPI-inositol deacylase localized to the ER. It removes an acyl chain on the inositol of GPI anchors after they are attached to proteins^48^. This modification is important for transport of GPI-APs from the ER to Golgi as demonstrated by PGAP1-deficient or –mutant cells, which have delayed ER-to-Golgi transport of GPI-APs^48–50^. Almost all GPI-APs are deacylated before their exit from the ER, and this modification generally occurs quickly after anchor attachment^15^. This process has been implicated in brain disorders given the association of PGAP1 mutations with intellectual disability and encephalopathy in humans^51–53^ as well as severe developmental defects in forebrain formation in mice^54–56^.

While no change in PGAP1 protein expression was observed in total homogenate, we found decreased expression of PGAP1 in an ER-enriched fraction in schizophrenia. Altered ER PGAP1 expression could result in abnormal deacylation of GPI-APs, leading to alterations in ER export, quality control, and degradation of a large class of important enzymes, receptors, and other biologically active membrane-bound proteins.

Once processing in the ER is complete, GPI-APs are recognized by p24 proteins and exported to the Golgi. The p24 family consists of type-I membrane proteins that are cycled between the ER and the Golgi by COP vesicles^57^. These families of proteins bind substrates and are packaged into COPII vesicles, facilitating substrate transport from the ER to the Golgi. Tmp21 is a p24 protein that specifically recognizes GPI-APs and is essential for GPI-AP quality control. We observed a decrease in Tmp21 protein expression in schizophrenia, suggesting abnormal regulation of export of GPI-APs from the ER.

Together, changes in PGAP1 and TMP21 expression implicate dysfunction in ER processing and export of GPI-APs. To examine potential functional consequences of these protein expression abnormalities, we separated membrane-bound and aqueous proteins and tested their sensitivity to PI-PLC, an enzyme that cleaves phosphatidylinositol. We used Triton X-114 phase partitioning, where amphiphilic proteins are recovered in the DT phase and soluble proteins partition to the AQ phase. Membrane-bound GPI-APs should partition to the DT phase, while unmodified forms of those substrates should be found in the AQ phase. Additionally, we treated the samples with and without PI-PLC to specifically remove the GPI anchor and thus result in GPI-APs partitioning to the AQ phase, allowing us to distinguish between the GPI modification and other methods of membrane-association. We examined expression of known GPI-APs previously associated with schizophrenia, GPC1, NCAM1, MDGA2, and EPHA1. We observed decreased sensitivity of DT phase-associated GPC1 and NCAM1, suggesting decreased expression of membrane-bound GPI-anchored GPC1 and NCAM1 in schizophrenia. This is consistent with dysfunction of GPI-AP ER export, which we propose based on decreased PGAP1 and Tmp21 expression. No change in PI-PLC sensitivity was observed in the expression of MDGA2 and EPHA1, suggesting that GPI-APs are differentially sensitive to the PGAP1 and Tmp21 expression abnormalities we found.

PI-PLC sensitivity or resistance is believed to represent the presence or absence, respectively, of a GPI-anchor^58,59^. However, resistance of some GPI-APs to release by PI-PLC arises from a change in the anchor structure via covalent modification of the inositol ring^60,61^. Therefore, an alternative interpretation of our findings is that rather than decreased expression of GPI-modified GPC1 and NCAM1, there could be an increase in abnormally modified forms of these GPI-APs. This may, in part, explain why despite the decrease in PI-PLC sensitive GPC1 and NCAM1 there is no change in membrane-associated expression of GPC1 and NCAM1; these proteins may have GPI anchors which allow them to associate with the membrane, but are not affected by PI-PLC treatment. In this case, the reduction in PGAP1 and TMP21 expression may represent faulty quality-control gating, rather than reflect limited export capacity. Quality control machinery prevents premature ER exit of folding intermediates and incompletely assembled proteins, which prevents partially or non-functional proteins from reaching terminal compartments where their presence could be toxic^62,63^. An example of the consequences of quality control dysregulation are neurodegenerative diseases, including Alzheimer’s Diseases, Parkinson’s Disease, Huntington’s Disease, Amyotrophic Lateral Sclerosis, and Creutzfeldt-Jakob disease, in which there is an accumulation of misfolded or unfolded proteins that results in neuropathological changes and eventual cell death^64^.

There are several limitations to consider when interpreting the current study. The subjects studied were elderly and the pathophysiology of subjects at late stages may not reflect abnormalities associated with earlier stages of the disorder. The majority of the schizophrenia subjects were receiving antipsychotic medication at time of death. To determine whether chronic antipsychotic treatment was sufficient to cause the observed abnormalities in protein expression and PI-PLC sensitivity, we repeated these studies in rats chronically treated with the antipsychotic haloperidol. No changes were observed, suggesting that our findings in schizophrenia are not likely due to chronic antipsychotic treatment. As proteins may differ in their stability over a range of PMI, we tested the effect of PMI on protein levels in mice. There were no significant effects of PMI on protein expression of Tmp21 or PGPA1 (53kD) in this study.

Our lab has previously reported abnormal immature N-linked glycosylation, an ER-specific protein modification, of glutamate and GABA system components in the frontal cortex in schizophrenia^9,10,17^. Recently, we demonstrated that while components of early N-glycosylation processing are intact, quality control mechanisms and ER associated degradation machinery are upregulated in the DLPFC of schizophrenia subjects, which may contributed to the abnormal expression of glycosylated proteins^13^. This pattern is consistent with what we found in the current study, where proteins regulating GPI-AP processing, quality control, and ER export are abnormally expressed. Together with our previous work, these findings support a growing body of evidence for a fundamental deficit in ER protein processing pathways. Our data illustrate the importance of ER associated protein processing and quality control machinery, and future work will be necessary to elucidate the mechanisms by which these abnormalities may contribute to the pathophysiology of schizophrenia.

## Supplementary information

Supplementary Table S1

Supplementary Table S2
