# Supplementary material for: Abnormal ER quality control of neural GPI-anchored proteins via dysfunction in ER export processing in the frontal cortex of elderly subjects with schizophrenia
Source: Transl Psychiatry. 2019 Jan 16;9:6. doi: 10.1038/s41398-018-0359-4 (PMC6341114; doi:10.1038/s41398-018-0359-4)
Supplement: Supplementary file 1 — Supplementary Table S1 [file 41398_2018_359_MOESM1_ESM.docx]

| **Supplementary Table S1.** Subject Demographics | | | | | | | |  | | | | |
| --- | --- | --- | --- | --- | --- | --- | --- | --- | --- | --- | --- | --- |
|  |  |  |  |  |  |  |  |  | | | | |
| **Pair** | **Subject** | **Rx** | **Sex/Age** | **Rin** | **pH** | **PMI (hr)** | **COD** | | **Analysis** | | |  |
| 1 | Schizophrenia | On | F/62 | 6.9 | 6.7 | 23.7 |  | | |  |  |  |
|  | Comparison |  | F/66 | 2.5 | 6.9 | 22.6 |  | | | P/F/S |  |  |
|  |  |  |  |  |  |  |  | | |  |  |  |
| 2 | Schizophrenia | Off | F/70 | 6.1 | 6.4 | 13.2 |  | | | P |  |  |
|  | Schizophrenia | Off | F/81 |  | 5.9 | 12.5 |  | | | F/S |  |  |
|  | Comparison |  | F/73 | 7.3 | 7 | 3 |  | | |  |  |  |
|  |  |  |  |  |  |  |  | | |  |  |  |
| 3 | Schizophrenia | On | F/75 | 4.6 | 6.5 | 21.5 |  | | |  |  |  |
|  | Comparison |  | F/74 | 6.1 | 6.3 | 4.8 | Cardio pulmonary arrest | | | P/F/S |  |  |
|  |  |  |  |  |  |  |  | | |  |  |  |
| 4 | Schizophrenia | On | F/77 | 2.7 | 6 | 9.7 | Cardiopulmonary arrest | | |  |  |  |
|  | Comparison |  | F/79 | 7.5 | 6.4 | 10.1 |  | | | P/F/S |  |  |
|  |  |  |  |  |  |  |  | | |  |  |  |
| 5 | Schizophrenia | Off | F/81 | 6.9 | 6.7 | 15.1 |  | | |  |  |  |
|  | Comparison |  | F/80 | 5.3 | 6.6 | 3.8 | Cardiopulmonary arrest | | | P |  |  |
|  | Comparison |  | F/81 |  | 6.4 | 19.4 |  | | | F/S |  |  |
|  |  |  |  |  |  |  |  | | |  |  |  |
| 6 | Schizophrenia | On | F/89 | 7.2 | 6.2 | 9.6 |  | | |  |  |  |
|  | Comparison |  | F/89 | 7.8 | 6.7 | 2.3 | Cardiopulmonary arrest | | | P/F/S |  |  |
|  |  |  |  |  |  |  |  | | |  |  |  |
| 7 | Schizophrenia | Off | F/84 |  | 6.8 | 21.9 |  | | |  |  |  |
|  | Comparison |  | F/85 |  | 7.3 | 8 |  | | | P/F/S |  |  |
|  |  |  |  |  |  |  |  | | |  |  |  |
| 8 | Schizophrenia | On | M/57 | 8.9 | 6.4 | 20.7 | Cardio respiratory failure | | | P |  |  |
|  | Schizophrenia | On | M/57 | 2.8 | 6.1 | 20.3 | Cardio respiratory arrest | | | F/S |  |  |
|  | Comparison |  | M/59 | 8.3 | 6.7 | 20.4 | Cardiopulmonary arrest | | |  |  |  |
|  |  |  |  |  |  |  |  | | |  |  |  |
| 9 | Schizophrenia | On | M/70 | 6.3 | 6.4 | 7.2 |  | | |  |  |  |
|  | Comparison |  | M/70 |  | 6.1 | 6.7 |  | | | P |  |  |
|  | Comparison |  | M/69 | 8.4 | 6.7 | 7.3 | Cardiac Arrest | | | F/S |  |  |
|  |  |  |  |  |  |  |  | | |  |  |  |
| 10 | Schizophrenia | On | M/73 | 4.6 | 6.5 | 7.9 | Cardio respiratory failure | | |  |  |  |
|  | Schizophrenia | On | M/68 |  | 6.8 | 5.6 | Cardio respiratory failure | | | F/S |  |  |
|  | Comparison |  | M/73 | 4.1 | 6.2 | 14.9 |  | | | P/F/S |  |  |
|  |  |  |  |  |  |  |  | | |  |  |  |
| 11 | Schizophrenia | On | M/78 | 3.7 | 6.6 | 9.4 |  | | |  |  |  |
|  | Comparison |  | M/75 | 7.7 | 6.4 | 5 |  | | | P |  |  |
|  | Comparison |  | M/68 | 7.1 | 6.6 | 2.8 |  | | | F/S |  |  |
|  |  |  |  |  |  |  |  | | |  |  |  |
| 12 | Schizophrenia | On | M/80 |  | 6.4 | 15.4 |  | | |  |  |  |
|  | Comparison |  | M/76 | 7 | 6.3 | 2.9 | Cardiopulmonary arrest | | | P/F/S |  |  |
|  |  |  |  |  |  |  |  | | |  |  |  |
| 13 | Schizophrenia | On | M/97 | 7.2 | 6.5 | 9.3 | Cardiopulmonary arrest | | |  |  |  |
|  | Comparison |  | M/95 | 6.8 | 6.5 | 4.1 | Cardio-resp. failure | | | P/S |  |  |
|  | Comparison |  | M/93 | 6.5 | 6.3 | 4.2 | Acute myocardial infarction | | | F |  |  |
|  |  |  |  |  |  |  |  | | |  |  |  |
| 14 | Schizophrenia | On | M/82 | 5.5 | 6.7 | 11.4 | Respiratory failure | | |  |  |  |
|  | Comparison |  | M/69 | 8.4 | 6.7 | 7.4 | Cardiac arrest | | | P/FS |  |  |
|  |  |  |  |  |  |  |  | | |  |  |  |
| 15 | Schizophrenia | On | M/92 | 7.4 | 6.7 | 17.7 |  | | |  |  |  |
|  | Comparison |  | M/93 | 6.5 | 6.3 | 4.2 | Acute myocardial infarction | | | P/F/S |  |  |
| Abbreviations: Male (M), Female (F), Postmortem Interval (PMI); Cause of death (COD); Rx: On = treated with antipsychotic medications at time of death; Off = off antipsychotic medication for > 6 weeks prior to death; P = Used in protein expression analysis, F= ER Fractionation expression analysis,S=Triton x-114 phase separation with PI-PLC expression analysis | | | | | | | | | | | | |
