# Supplementary material for: Abnormal ER quality control of neural GPI-anchored proteins via dysfunction in ER export processing in the frontal cortex of elderly subjects with schizophrenia
Source: Transl Psychiatry. 2019 Jan 16;9:6. doi: 10.1038/s41398-018-0359-4 (PMC6341114; doi:10.1038/s41398-018-0359-4)
Supplement: Supplementary file 2 — Supplementary Table S2 [file 41398_2018_359_MOESM2_ESM.docx]

| **Supplementary Table S2.** Antibodies Used for Western Blot Analysis | | | | |
| --- | --- | --- | --- | --- |
| Target Protein | Host | Dilution | Buffer | Vendor (cat.#) |
| GPAA1 | Rabbit | 1:500 | Li-cor | Genetex (GTX115131) |
| Tmp21 | Mouse | 1:1,000 | Licor | Proteintech (15199-1-AP) |
| PGAP1 | Mouse | 1:1,000 | Li-cor | Proteintech (55392-1-AP) |
| NCAM1 | Goat | 1:2,000 | 50% Licor | Novus Biologicals (AF2408) |
| MDGA2 | Rabbit | 1:500 | 50% Licor | Abcam (ab135407) |
| GPC1 | Rabbit | 1:1,000 | 50% Licor | Genetex (GTX104557) |
| EPHA1 | Rabbit | 1:500 | 50% Licor | Abcam (ab65072) |
| JM4 | Mouse | 1:2,000 | Li-cor | Abcam (ab53113) |
| VCP | Rabbit | 1:2,000 | Li-cor | Abcam (ab11740) |
| VCP | Mouse | 1:1,000 | Li-cor | Abcam (ab11433) |
| 50 % buffers were diluted to the indicated concentration with Tris-buffered saline with 0.1% Tween-20 Abbreviations: GPI anchor attachment 1 (GPAA1); type-I membrane proteins (Tmp21); post-GPI attachment to proteins 1 (PGAP1); neural cell adhesion molecule (NCAM); MAM domain-containing GPI anchor protein 2 (MDGA2); Glypican1 (GPC1); Ephrin type A receptor (EPHA1); Jena-Muenchen4 (JM4); Valosin containing protein (VCP); Li-core Odyssey blocking buffer (Li-cor). | | | | |
